# Overexpression of Activation-Induced Cytidine Deaminase in MTX- and Age-Related Epstein-Barr Virus-Associated B-Cell Lymphoproliferative Disorders of the Head and Neck

**DOI:** 10.1155/2015/605750

**Published:** 2015-03-05

**Authors:** Kentaro Kikuchi, Toshiyuki Ishige, Fumio Ide, Yumi Ito, Ichiro Saito, Miyako Hoshino, Harumi Inoue, Yuji Miyazaki, Tadashige Nozaki, Masaru Kojima, Kaoru Kusama

**Affiliations:** ^1^Division of Pathology, Department of Diagnostic and Therapeutic Sciences, Meikai University School of Dentistry, 1-1 Keyakidai, Sakado, Saitama 350-0283, Japan; ^2^Department of Pathology, Nihon University School of Medicine, 30-1 Oyaguchi-Kamimachi, Itabashi-ku, Tokyo 173-8610, Japan; ^3^Division of Diagnostic Pathology, Tsurumi University Dental Hospital, 2-1-3 Tsurumi, Tsurumi-ku, Yokohama 230-8501, Japan; ^4^Department of Pathology, Tsurumi University School of Dental Medicine, 2-1-3 Tsurumi, Tsurumi-ku, Yokohama 230-8501, Japan; ^5^Second Division of Oral and Maxillofacial Surgery, Department of Diagnostic and Therapeutic Sciences, Meikai University School of Dentistry, 1-1 Keyakidai, Sakado, Saitama 350-0283, Japan; ^6^Department of Pharmacology, Osaka Dental University, 8-1 Kuzuhahanazono-cho, Hirakata, Osaka 573-1211, Japan; ^7^Department of Anatomic and Diagnostic Pathology, Dokkyo Medical University School of Medicine, 880 Oaza-kitakobayashi, Mibu-machi, Shimotsuga-gun, Tochigi 321-0293, Japan

## Abstract

Recent research has shown that activation-induced cytidine deaminase (AID) triggers somatic hypermutation and recombination, in turn contributing to lymphomagenesis. Such aberrant AID expression is seen in B-cell leukemia/lymphomas, including Burkitt lymphoma which is associated with *c-myc* translocation. Moreover, Epstein-Barr virus (EBV) latent membrane protein-1 (LMP-1) increases genomic instability through early growth transcription response-1 (Egr-1) mediated upregulation of AID in B-cell lymphoma. However, few clinicopathological studies have focused on AID expression in lymphoproliferative disorders (LPDs). Therefore, we conducted an immunohistochemical study to investigate the relationship between AID and LMP-1 expression in LPDs (MTX-/Age-related EBV-associated), including diffuse large B-cell lymphomas (DLBCLs). More intense AID expression was detected in LPDs (89.5%) than in DLBCLs (20.0%), and the expression of LMP-1 and EBER was more intense in LPDs (68.4% and 94.7%) than in DLBCLs (10.0% and 20.0%). Furthermore, stronger Egr-1 expression was found in MTX/Age-EBV-LPDs (83.3%) than in DLBCLs (30.0%). AID expression was significantly constitutively overexpressed in LPDs as compared with DLBCLs. These results suggest that increased AID expression in LPDs may be one of the processes involved in lymphomagenesis, thereby further increasing the survival of genetically destabilized B-cells. AID expression may be a useful indicator for differentiation between LPDs and DLBCLs.

## 1. Introduction

Epstein-Barr virus (EBV) is associated with a variety of lymphoproliferative disorders (LPDs) and other malignancies [1–5], including nasopharyngeal carcinoma, Hodgkin disease, and Burkitt lymphoma [6–9]. EBV-driven B-cell LPDs can be age-related or can occur in patients who are immunosuppressed due to primary immune deficiency, HIV infection, organ transplantation, and treatment with methotrexate or tumor necrosis factor-*α* antagonist for rheumatoid arthritis [10, 11]. The major EBV oncogene, latent membrane protein-1 (LMP-1), activates signaling pathways such as those involving nuclear factor-kappa-light-chain-enhancer of activated B-cells (NF-*κ*B), which enhances B-cell survival and is essential for EBV-induced transformation [12–16]. LMP-1 is a 63 kDa integral membrane protein with three domains and contains two distinct functional regions within its C-terminus, designated C-terminal activating regions 1 and 2 (CTAR1 and CTAR2). The protein also protects cancer cells from apoptosis, by inducing antiapoptotic proteins, including BCL-2, MCL-1, A20, early growth response transcription factor-1 (Egr-1), and SNARK [17–19]. Recent studies have shown that EBV-infected cells undergo hypermutation or switching of recombination* in vivo* via upregulation of activation-induced cytidine deaminase (AID) [20] and also that EBV-induced AID is associated with oncogene mutations, which contribute to lymphomagenesis [21]. The relationship between LMP-1 and cancer has been relatively well established, while the molecular mechanisms underlying AID induction remain to be fully clarified.

AID is normally expressed in germinal center (GC) B-cells [22], where it plays a central role in both somatic hypermutation and class switch recombination in humans and mice [23, 24]. AID converts single-stranded genomic cytidine into uracil, with pronounced activity in the immunoglobulin variable and switch regions [25–28]. Aberrant expression of AID and abnormal targeting of AID activation in both B- and non-B-cells cause DNA double-strand breaks (DSBs) and DNA point mutations in both Ig and non-Ig genes, inducing tumorigenesis [29]. AID is required for chromosomal DSBs at the* c-myc* and* IgH* loci, which lead to reciprocal* c-myc/IgH* translocations, resulting in the development of B-cell lymphomas, such as Burkitt lymphoma in humans and plasmacytoma in mice [30]. AID protein is localized more in the cytoplasm than in the nucleus in normal and neoplastic B-cells, and cytoplasmic AID protein relocates to the nucleus when pathological change occurs in B-cells [31, 32].

A recent* in vitro *study by Kim et al. [33] has shown that LMP-1 increases genomic instability through Egr-1-mediated upregulation of AID in B-cell lymphoma cell lines. However, to our knowledge, no clinicopathological case study has examined the expression of LMP-1, AID, and Egr-1, including the distribution and density of positive cells, on LPDs. It is therefore important to clarify the expression pathway and distribution of positive cells in lesions of human tissues. We considered that AID positive cells would be more numerous in the EBV-driven LPDs than in DLBCLs showing a monotonous growth pattern. Therefore, we conducted an immunohistochemical study to investigate the relationship between LMP-1, AID, and Egr-1 expression in LPDs (MTX-/Age-related EBV-associated), including DLBCLs.

## 2. Materials and Methods

### 2.1. Tissue Samples

A total of 29 biopsy specimens were retrieved from the three hospitals to which the authors have contributed pathological diagnosis and were presented for investigation. Tissue samples from 17 cases of MTX-EBV-LPD, 2 cases of Age-EBV-LPD, and 10 cases of DLBCL were used (Table 1). Sporadic-Burkitt lymphoma (sBL) [34], MTX-LPD [35], Age-LPD [36], and oral squamous cell carcinoma (OSCC) were used as an overexpressing positive control for AID, LMP-1, EBV-encoded small RNA (EBER), and Egr-1. Ten samples of cervical lymph nodes (LNs) showing reactive lymphoid hyperplasia (RLH) were used as normal positive controls for AID, LMP-1, EBER, and Egr-1. Each section was prepared for immunohistochemical analysis (IHC) and* in situ* hybridization (ISH). The case study protocol was reviewed and approved by the Research Ethics Committee of Meikai University School of Dentistry (A0832, A1321).

### 2.2. Immunohistochemistry

Deparaffinized sections were immersed for 15 min at room temperature in absolute methanol containing 0.3% H_2_O_2_ to block endogenous peroxidase activity and then treated with 2% bovine serum albumin for 15 min to block nonspecific reactions. After washing, they were incubated with an appropriately diluted mouse monoclonal antibody against human LMP-1 and rabbit polyclonal antibodies against AID and Egr-1 (Table 2). After washing, the sections were incubated with a prediluted anti-mouse or rabbit IgG antibody conjugated with peroxidase (Nichirei, Tokyo, Japan) for 30 min at room temperature. They were then immersed for 8 min in 0.05% 3,3′-diaminobenzidine tetrahydrochloride (DAB) in 0.05 M Tris-HCl buffer (pH 8.5) containing 0.01% H_2_O_2_ and counterstained with Mayer's haematoxylin for 90 s.

### 2.3. *In Situ* Hybridization

ISH for EBER oligonucleotides was performed to detect the presence of EBV small RNA in formalin-fixed paraffin-embedded sections using a hybridization kit (Dako, A/S, Denmark) in accordance with the manufacturer's instructions. Age-EBV-LPD was used as a positive control for EBER [36].

### 2.4. Assessment of AID, LMP-1, EBER, and Egr-1 Expression in Biopsy Specimens

Reactivity for each of the antigens and EBER was evaluated semiquantitatively using a light microscope (model BH2, Olympus Corp.). The distribution of the staining was categorized semiquantitatively according to the ratio of the positive area as follows: diffuse (+++) ≧75%; focal (++) <75% to ≧25%; partial (+) <25% to ≧5%; few (−/±) negative/<5% or nonspecific. The intensity of the staining was categorized semiquantitatively as strong (S), moderate (M), weak (W), or negative (N) relative to each control specimen. AID intensity was compared with that in the sBL case sample used as a positive control [34] and expressed as strong (S) when higher or of the same intensity as that in sBL, moderate (M) when lower than that in sBL or of the same intensity as that in RLH, weak (W) when lower than that in RLH, and negative (N) in case of no staining or nonspecific staining. LMP-1 and EBER intensity were compared with those in MTX-LPD and Age-EBV-LPD used as a positive control [35, 36] and evaluated in the same manner as those for AID. Egr-1 intensity was compared with that in OSCC used as a positive control and evaluated in the same manner as that for AID.

### 2.5. Statistical Analysis

The significance of differences between the mean values was determined by using the Mann-Whitney *U* test or Exact Binominal test for comparing two categories. The accepted level of significance was *P* < 0.05.

## 3. Results

**Figure 1:**
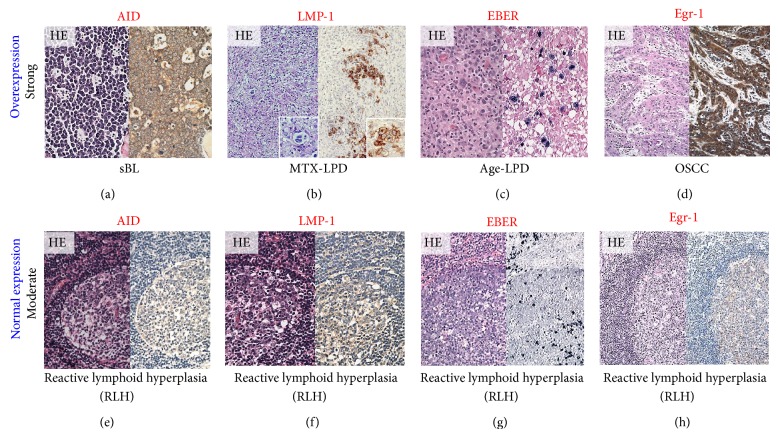
Positive control for AID, LMP-1, EBER, and Egr-1 in the overexpression and normal expression. (a) Sporadic Burkitt lymphoma (sBL) [34], (b) MTX-LPD [35], (c) Age-LPD [36], and (d) oral squamous cell carcinoma (OSCC) were used as an aberrant positive control (strong intensity/overexpression) for AID, LMP-1, EBER, and Egr-1 (hematoxylin-eosin staining: HE, left panel; immunohistochemical staining: IHC, right panel) ((a)–(d) original magnification ×100). Reactive lymphoid hyperplasia (RLH) was used as normally positive control (moderate/normal expression) for (e) AID, (f) LMP-1, (g) EBER, and (h) Egr-1 ((e)–(h) original magnification ×100). (g) Germinal center B-cells were moderate/normal positive for EBER, and plasma cells were strongly positive for EBER (right panel).

**Figure 2:**
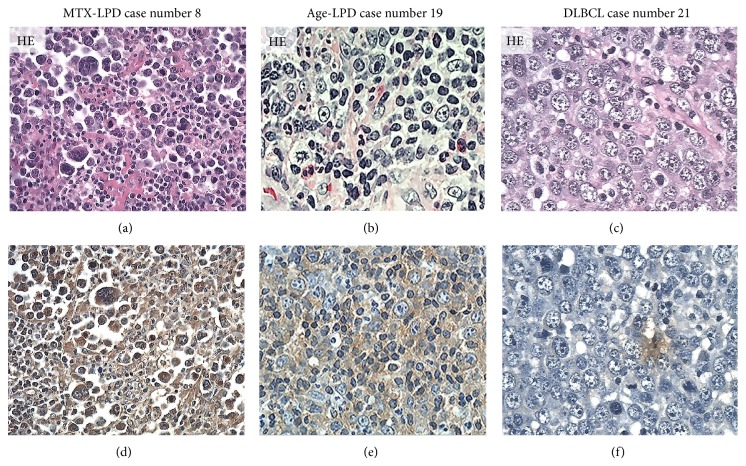
Distribution and intensity of AID expression in MTX-/Age-EBV-LPDs and DLBCLs in biopsy specimens. ((a)–(c)) HE stain and ((d)–(f)) AID by IHC (brownish color). AID positive atypical lymphoid cells were diffuse in (d) MTX-LPD and (e) Age-LPD but were few in (f) DLBCL. AID positive cells were of strong intensity in (d) MTX-LPD and (e) Age-LPD and were of (f) weak or moderate intensity in DLBCL ((a)–(f) original magnification, ×200).

**Figure 3:**
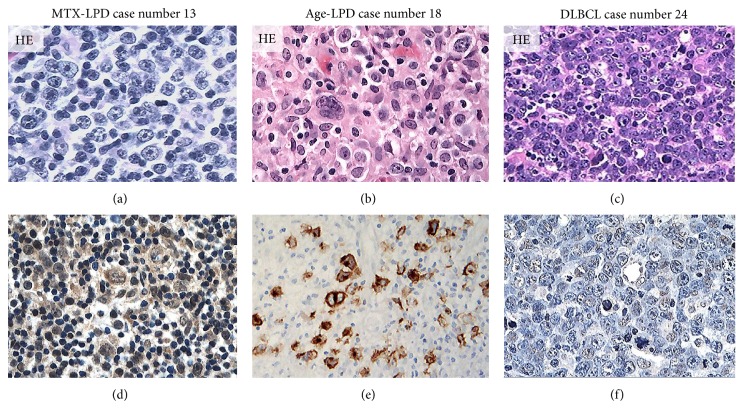
Distribution and intensity of LMP-1 expression in MTX-/Age-EBV-LPDs and DLBCLs in biopsy specimens. ((a)–(c)) HE stain and ((d)–(f)) LMP-1 by IHC. LMP-1 positive atypical lymphoid cells (brownish color) were diffuse or sporadic diffuse in (d) MTX-LPD or (e) Age-LPD but were not verifiable in (f) DLBCL. LMP-1 positive cells were of moderate or strong intensity in (d) MTX-LPD and (e) Age-LPD and were of weak or nonspecific intensity in (f) DLBCL ((a)–(f) original magnification, ×200).

**Figure 4:**
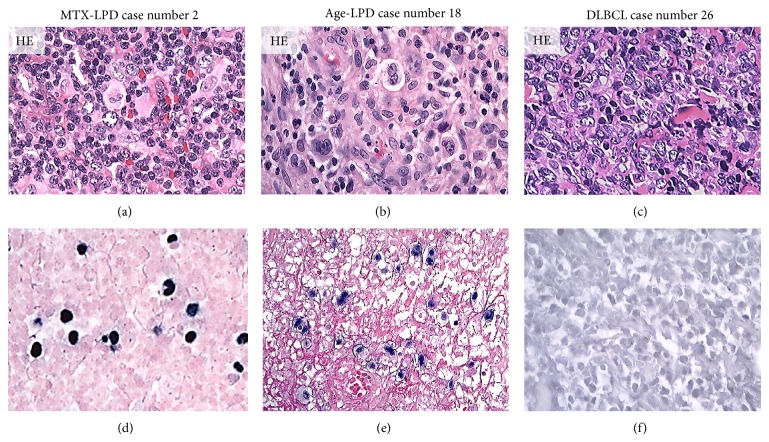
Distribution and intensity of EBER expression in MTX-/Age-EBV-LPDs and DLBCLs in biopsy specimens. ((a)–(c)) HE stain and ((d)–(f)) EBER by ISH (blackish color). EBER positive atypical lymphoid cells were sporadic diffuse in (d) MTX-LPD and (e) Age-LPD but were not verifiable in (f) DLBCL. EBER positive cells were of almost strong intensity in (d) MTX-LPD and (e) Age-LPD and were of weak or nonspecific intensity in (f) DLBCL ((a)–(f) original magnification, ×200).

Strong AID, LMP-1, EBER, and Egr-1 reactivity were observed in overexpressing positive control specimens in sBL, MTX-LPD, Age-LPD, and OSCC by IHC and ISH (Figures 1(a)–1(d)). Moderate AID, LMP-1, EBER, and Egr-1 reactivity were observed in normal positive control specimens RLM by IHC and ISH (Figures 1(e)–1(h)). AID expression was diffuse and strongly positive in MTX-/Age-EBV-LPDs (Figures 2(a), 2(b), 2(d), and 2(e)) and was few and moderately positive in DLBCLs (Figures 2(c) and 2(f)). Although LMP-1 expression was diffuse and strongly positive in MTX-/Age-EBV-LPDs (Figures 3(a), 3(b), 3(d), and 3(e)), the expression was few and weakly positive in DLBCLs (Figures 3(c) and 3(f)). EBER expression was sporadic diffuse and strongly positive in MTX-/Age-EBV-LPDs (Figures 4(a), 4(b), 4(d), and 4(e)), while EBER reactivity was negative in DLBCLs (Figures 4(c) and 4(f)). Expression of AID, LMP-1, and EBER was higher in MTX-/Age-EBV-LPDs than in DLBCLs. Staining patterns, AID, LMP-1, and EBER, were compared between different lesion types (Table 1).

**Figure 5:**
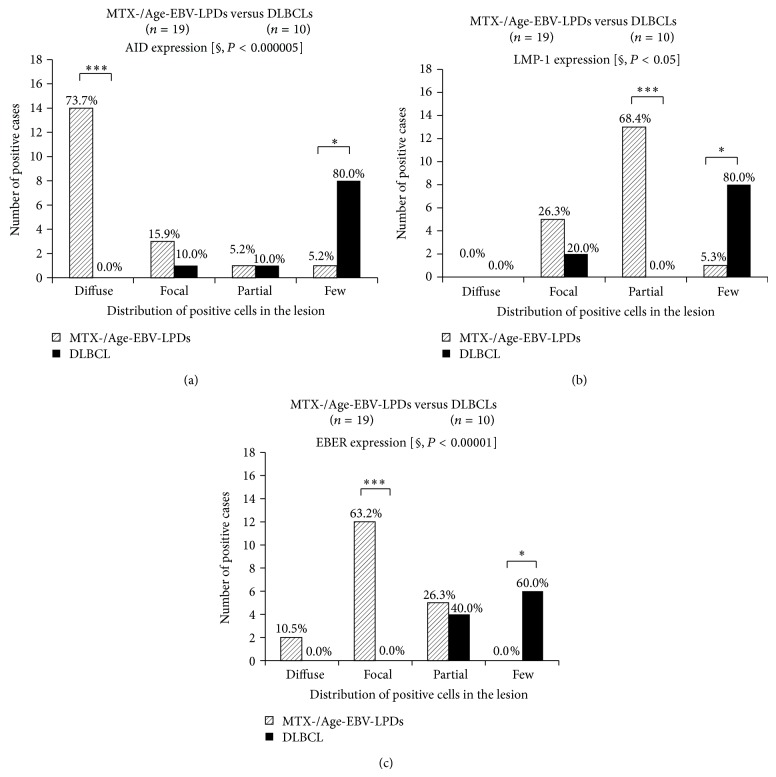
Distribution of AID, LMP, and EBER expression in MTX-/Age-EBV-LPDs and DLBCLs. ((a), (b), and (c)) The distribution of positive cells for AID, LMP, and EBER was more extensive in MTX-/Age-EBV-LPDs than in DLBCLs. *P* values were examined by Mann-Whitney *U* test [§] or Exact Binominal test (^*^ *P* < 0.05, ^**^ *P* < 0.01, and ^***^ *P* < 0.001).

**Figure 6:**
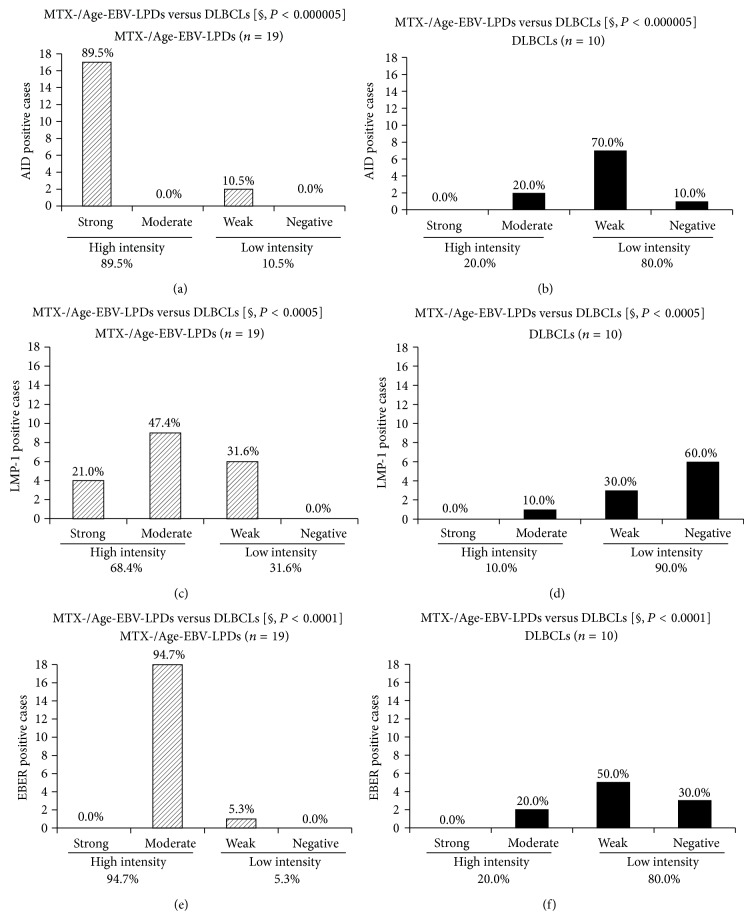
Intensity of AID, LMP-1, and EBER expression in MTX-/Age-EBV-LPDs and DLBCLs. High intensity rate of AID was higher in (a) MTX/Age-EBV-LPDs (89.5%) than in (b) DLBCLs (20.0%). Conversely, low intensity rate of AID was higher in (b) DLBCLs (80.0%) than in (a) MTX/Age-EBV-LPDs (10.5%). *P* < 0.000005 by Mann-Whitney *U* test [§]. High intensity rate of LMP-1 was higher in (c) MTX/Age-EBV-LPDs (68.4%) than in (d) DLBCLs (10.0%). Conversely, low intensity rate of LMP-1 was higher in (d) DLBCLs (90.0%) than in (c) MTX/Age-EBV-LPDs (31.6%). *P* < 0.0005 by Mann-Whitney *U* test [§]. High intensity rate of EBER was higher in (e) MTX/Age-EBV-LPDs (94.7%) than in (f) DLBCLs (20.0%). Conversely, low intensity rate of EBER was higher in (f) DLBCLs (80.0%) than in (e) MTX/Age-EBV-LPDs (5.3%). *P* < 0.0001 by Mann-Whitney *U* test [§].

**Figure 7:**
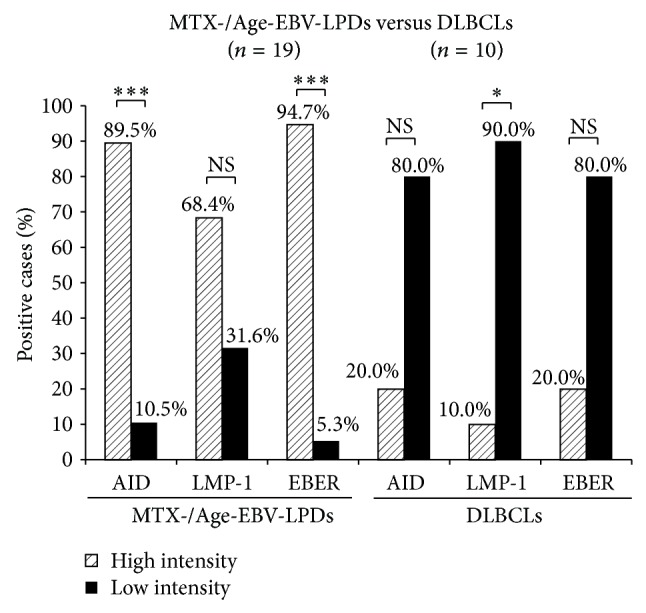
Comparison of the intensity of AID, LMP-1, and EBER expression between MTX/Age-EBV-LPDs and DLBCLs. High intensity showed strongly and moderately positive cases, and low intensity showed weakly positive and negative cases. Although a high intensity of expression (AID, LMP-1, and EBER) was greater in MTX-/Age-EBV-LPDs than in DLBCLs, a low intensity of those was smaller in the former than in the latter. *P* values were examined by Exact Binominal test (^*^ *P* < 0.05, ^**^ *P* < 0.01, and ^***^ *P* < 0.001). NS: not significant.

**Figure 8:**
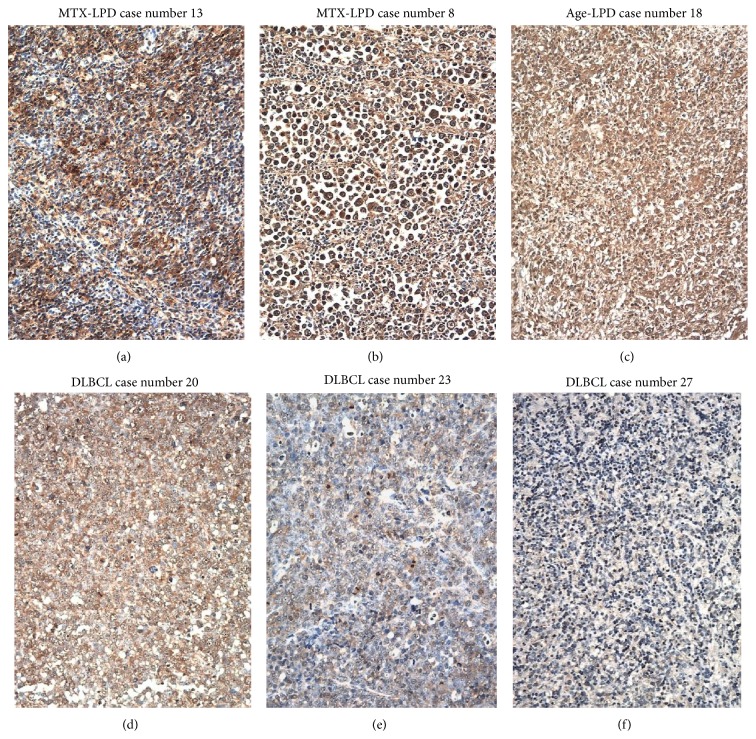
Distribution and intensity of Egr-1 expression in MTX-/Age-LPDs and DLBCLs in biopsy specimens. Egr-1 positive cells (brownish color) were diffuse and of strong intensity in ((a), (b)) MTX-LPDs and (c) Age-LPD and were a variety in ((d)–(f)) DLBCLs ((a)–(f) original magnification ×100).

**Figure 9:**
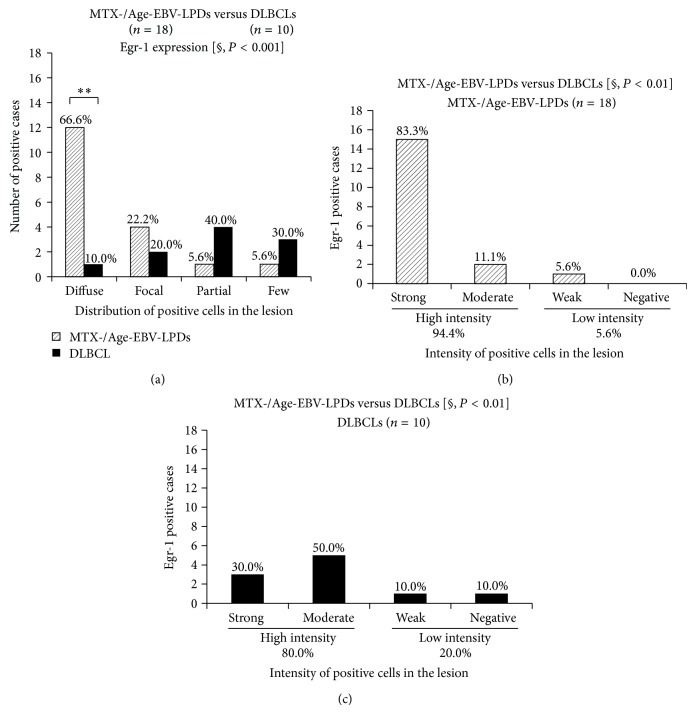
Distribution and intensity of Egr-1 expression in MTX-/Age-EBV-LPDs and DLBCLs. (a) Distribution of Egr-1-positive cells was more extensive in MTX-/Age-EBV-LPDs than in DLBCLs (*P* < 0.001). (a) In diffuse category, distribution of Egr-1 was significantly greater in MTX-/Age-EBV-LPDs (66.6%) than in DLBCLs (10.0%). ((b), (c)) Although the intensity of Egr-1 expression was high in both MTX/Age-EBV-LPDs (94.4%) and DLBCLs (80.0%), in the strong category, it was greater in the former (83.3%) than in the latter (30.0%). *P* values were examined by Mann-Whitney *U* test [§] or Exact Binominal test (^*^ *P* < 0.05, ^**^ *P* < 0.01, and ^***^ *P* < 0.001).

The distribution of AID (*P* < 0.000005), LMP-1 (*P* < 0.05), and EBER (*P* < 0.00001) expression was significantly more extensive in the MTX-/Age-EBV-LPDs than in the DLBCLs (Figures 5(a)–5(c)). In addition, AID expression was significantly more intense in MTX-/Age-EBV-LPDs than in DLBCLs (*P* < 0.000005) (Figures 6(a) and 6(b)), and expression of LMP-1 (*P* < 0.0005) (Figures 6(c) and 6(d)) and EBER (*P* < 0.0001) was more intense in MTX-/Age-EBV-LPDs than in DLBCLs (Figures 6(e) and 6(f)). The high intensity (strong and moderate) of AID, LMP-1, and EBER expression was greater in MTX/Age-EBV-LPDs (89.5%, 68.4%, and 94.7%) than in DLBCLs (20.0%, 10.0%, and 20.0%) (Figures 6 and 7). Conversely, the low intensity (weak and negative) of AID, LMP-1, and EBER expression was greater in DLBCLs (80.0%, 90.0%, and 80.0%) than in MTX/Age-EBV-LPDs (10.5%, 31.6%, and 5.3%) (Figures 6 and 7). In MTX-/Age-EBV-LPDs, the intensity of AID, LMP-1, and EBER expression was stronger than in DLBCLs (Figure 7). Egr-1 expression was diffuse and strongly positive in MTX-/Age-EBV-LPDs (Figures 8(a)–8(c)) and was a positive variety in the DLBCLs (Figures 8(d)–8(f)). Distribution of Egr-1 expression was significantly more extensive in MTX-/Age-EBV-LPDs than in the DLBCLs (*P* < 0.001) (Figure 9(a)). The intensity of Egr-1 was significantly different between MTX-/Age-EBV-LPDs and DLBCLs (*P* < 0.01) (Figures 9(b) and 9(c)). Although the high intensity of Egr-1 expression was comparable rate in both MTX-/Age-EBV-LPDs (94.4%) and DLBCLs (80.0%), strong intensity was higher in MTX-/Age-EBV-LPDs (83.3%) (Figure 9(b)) than in DLBCLs (30.0%) (Figure 9(c)).

## 4. Discussion 

Immunohistochemical analysis in this study revealed that the expression of AID, LMP-1, and Egr-1 had a much more diffuse distribution and was stronger in intensity in LPD than in DLBCL cases. Furthermore, LPD cases showed a more diffuse distribution and stronger intensity of EBER-ISH than DLBCL cases.

EBV is associated with a variety of LPDs and malignant lymphomas [1, 3–5]. EBV-driven B-cell LPDs occur in patients who are immunosuppressed due to primary immune deficiency, HIV infection, or organ transplantation or patients who have received other treatments including methotrexate and tumor necrosis factor-*α* antagonists [10, 11]. Primary EBV infection is usually asymptomatic and leads to latent infection in memory B-cells, which do not permit viral replication [37]. Although newly infected naive B-cells have the phenotypes of transformed cells, they are controlled by both EBV-specific cytotoxic T lymphocytes and natural killer cells unless immunity is suppressed [37, 38]. In immunocompromised hosts, transformed cells become proliferating blasts that can result in symptomatic disease, such as immunodeficiency-associated LPD [1, 10, 37, 38]. LPD is characterized pathologically by focal or diffuse proliferation of atypical large B-cells including Reed-Sternberg-like cells with reactive components, which pose a diagnostic problem for pathologists. The spectrum of EBV-LPD is broad, ranging from benign polyclonal reactivation lesions to monoclonal EBV-DLBCL [39].

The major EBV-encoded LMP-1 is an integral membrane protein, which activates signaling pathways such as that involving NF-*κ*B, which increases B-cell survival and induces transformation [12–16] by inducing antiapoptotic protein [17–19]. An* in vitro* study has reported that EBV-infected cells undergo hypermutation or switching of recombination via AID upregulation [20], and EBV-induced AID is also associated with oncogene mutations, which contribute to lymphomagenesis [21]. In a mouse bone marrow transplantation model, AID overexpression was reported to promote B-cell lymphomagenesis [40]. Although the relationship between LMP-1 and lymphomagenesis has been relatively well established, the molecular mechanisms underlying AID induction remain to be fully clarified. Recently, Kim et al. have reported that LMP-1 increases genomic instability through Egr-1-mediated upregulation of AID in B-cell lymphoma [18]. The Egr-1 gene (also named zif268, NGFI-A, or Krox24) encodes an 80 kDa DNA-binding transcription factor [41]. Egr-1 is an exceptionally multifunctional transcription factor. In response to growth factors and cytokine signaling, Egr-1 regulates cell growth, differentiation, and apoptosis [42]. Egr-1 has been associated with EBV infection, a human gamma herpes virus closely associated with several lymphoid and epithelial malignancies [43]. First, Egr-1 is upregulated when EBV interacts with B lymphocytes at the initial infection stage, and constitutive expression of Egr-1 correlates with certain types of EBV latency in B-lymphoid cell lines [44]. EBV reactivation is associated with upregulation of Egr-1, and Egr-1 can be induced as an EBV lytic transactivator [45]. However, there are no reports of any clinicopathological studies on LMP-1, AID, and Egr-1 in samples of human tissue. Therefore, we examined the density and distribution of AID, LMP-1, EBER, and Egr-1 in 19 cases of LPD and 10 cases of DLBCL.

The distribution of AID, LMP-1, and EBER expression was more extensive in patients with LPD than in patients with DLBCL. The intensity of AID, LMP-1, and EBER expression was higher in LPD (89.5%, 68.4%, and 94.7%) than in DLBCL (20.0%, 10.0%, and 20.0%) patients (Figure 7). Although a higher intensity of expression was seen in LPD (94.4%) and DLBCL (80%), the intensity of Egr-1 expression was stronger in the former (83.3%) than in the latter (30.0%) (Figures 9(b) and 9(c)). These* in vivo* results partly supported the previous* in vitro* study by Kim et al. [18] and suggest that overexpression of AID in LPDs may be one process in the course of tumorigenic transformation.

The factor responsible for the lack of lymphoid tissue involvement in oral areas in patients with primary lymphoma/LPD is unclear, but there may be some association with bacteria in and around the teeth, together with chronic inflammation such as apical and marginal periodontitis. The copy number of EBV-DNA in subgingival plaque is associated with the presence of some periodontal bacteria [46]. A recent study has shown that periodontal disease could act as a risk factor for HIV reactivation [47] and similarly induce EBV reactivation [48]. Thus, there may be a relationship between AID, LMP-1, and Egr-1 expression in EBV-infected B-cells. Further studies, including the head and neck, will be needed to confirm the causal link between oral bacteria and EBV-positive lymphoma/LPDs of the oral cavity.

These results suggest that increased AID expression in LPDs may be part of the process of lymphomagenesis, thereby further increasing the survival of genetically destabilized B-cells. The reason why AID, LMP-1, and EBER were expressed more in the EBV related LPDs compared to DLBCL could be either the EBV infection or immunosuppression that is predominant in age-related lymphoma or in autoimmune diseases of patients taking methotrexate. The intensity and distribution of AID expression may be an indicator for differentiating EBV-driven LPDs from DLBCLs.

## Figures and Tables

**Table 1:** Case mix of patient with MTX-/Age-EBV-LPDs and DLBCLs in the head and neck.

Case number	Diagnosis	Age (y)/sex	Biopsy site	Collagen disease	Treatment of collagen disease	Histological type	ISH and immunohistochemistry			
							EBER-ISH	LMP-1	AID	Egr-1
1	MTX-LPD	32/F	LN (neck/axilla)	RA	MTX	NA	+ (M)	+ (W)	+++ (S)	+++ (S)
2	MTX-LPD	57/M	LN (neck)	RA	MTX	LPL	+++ (M)	+ (M)	+++ (S)	++ (M)
3	MTX-LPD	73/F	Oral mucosa (?)	RA	MTX/PSL	LPL	++ (M)	+ (M)	+ (W)	NA
4	MTX-LPD	52/M	LN (neck)	RA	MTX	HDMC	++ (M)	++ (W)	+++ (S)	+++ (S)
5	MTX-LPD	57/F	LN (left neck and axilla)	RA	MTX/PSL	HDNS	+++ (M)	+ (S)	+++ (S)	+++ (S)
6	MTX-LPD	71/F	Septonasal mucosa	RA	MTX	NA	++ (M)	+ (M)	+++ (S)	+++ (S)
7	MTX-LPD	60/F	LN (neck)	RA	MTX/PSL	LPL	++ (M)	+ (M)	+++ (S)	−/± (W)
8	MTX-LPD	64/M	LN (neck)	RA	MTX/PSL	DLBCL	++ (M)	+ (M)	+++ (S)	+++ (S)
9	MTX-LPD	44/M	Gingiva (right upper)	RA (Still's disease)	MTX/PSL	DLBCL	++ (M)	+ (M)	+++ (S)	++ (S)
10	MTX-LPD	69/F	Gingiva (left upper)	RA	MTX	HD-like	++ (M)	+ (M)	+++ (S)	+++ (S)
11	MTX-LPD	76/F	Gingiva (right upper)	RA	MTX	HD-like	++ (M)	+ (M)	+++ (S)	+++ (S)
12	MTX-LPD	67/M	Hard palate (midline)	RA	MTX	NA	+ (M)	−/±, (W)	−/± (W)	+ (M)
13	MTX-LPD	67/M	LN (neck)	RA	MTX/PSL	Follicular	++ (M)	+ (W)	++ (S)	++ (S)
14	MTX-LPD	56/F	LN (left neck)	RA	MTX/PSL	MALT	+ (M)	+ (W)	+++ (S)	+++ (S)
15	MTX-LPD	64/F	Skin (?)	RA	MTX/PSL	DLBCL	++ (M)	++ (S)	++ (S)	+++ (S)
16	MTX-LPD	59/M	Thyroid gland	RA	MTX/PSL	DLBCL	++ (M)	++ (W)	++ (S)	+++ (S)
17	MTX-LPD	74/F	Parotid gland (left)	RA	MTX/PSL	DLBCL	+ (M)	++ (S)	+++ (S)	++ (S)
18	Age-LPD	71/M	Mandibular bone (intraosseous)	N	NT	Polymorphous	++ (S)	++ (S)	+++ (S)	+++ (S)
19	Age-LPD	76/M	Tongue/floor of mouth (right)	N	NT	Intermediate, DLBCL/CHL	+ (W)	+ (M)	+++ (S)	+++ (S)
20	DLBCL	78/M	Maxillary sinus (right)	N	NT	DLBCL	−/± (W)	++ (M)	−/± (W)	+++ (S)
21	DLBCL	63/M	LN (left submandibular)	N	NT	DLBCL	+ (W)	−/± (N)	−/± (W)	−/± (M)
22	DLBCL	64/M	Gingiva (upper)	N	NT	DLBCL	+ (W)	−/± (N)	−/± (W)	−/± (M)
23	DLBCL	82/F	Gingiva (right lower)	N	NT	DLBCL	−/± (N)	++ (W)	++ (M)	++ (S)
24	DLBCL	69/F	Gingiva (left upper)	N	NT	DLBCL	+ (M)	−/± (W)	−/± (W)	+ (M)
25	DLBCL	56/M	Gingiva (right upper)	N	NT	DLBCL	+ (M)	−/± (W)	+ (M)	+ (S)
26	DLBCL	52/F	Gingiva (upper)	N	NT	DLBCL	−/± (N)	−/± (N)	−/± (W)	+ (W)
27	DLBCL	46/M	Gingiva (left lower)	N	NT	DLBCL	−/± (W)	−/± (N)	−/± (N)	−/± (N)
28	DLBCL	70/F	LN (left neck)	N	NT	DLBCL	−/± (W)	−/± (N)	−/± (W)	+ (M)
29	DLBCL	71/F	Maxillary sinus (left)	N	NT	DLBCL	−/± (N)	−/± (N)	−/± (W)	++ (M)

MTX: methotrexate; LPD: lymphoproliferative disorder; DLBCL: diffuse large B-cell lymphoma; RA: rheumatoid arthritis; LPL: lymphoplasmacytic lymphoma; HDMC: Hodgkin disease, mixed cellularity; HDNS: Hodgkin disease, nodular sclerosis; MALT: mucosa-associated lymphoid tissue; CHL: classical Hodgkin lymphoma; HD-like: Hodgkin disease-like; NA: not available; NT: no treatment; PSL: prednisolone; ?: unknown; LN: lymph node; LMP-1: Epstein-Barr virus- (EBV-) latent infection membrane protein-1; ISH: *in situ* hybridization; EBER: EBV-encoded small RNA; AID: activation-induced cytidine deaminase; Egr-1: early growth response transcription factor-1; diffuse: +++ (≧75%); focal: ++ (<75% to ≧25%); partial: + (<25% to ≧5%); few: −/± (negative/<5% or nonspecific); (S): strongly positive; (M): moderately positive; (W): weakly positive; (N): negative; M: male; F: female.

**Table 2:** Antibodies and dilutions used in this study.

Antigen	Clone	Dilution	Pretreatment	Primary antibody incubation time	Source
AID	—	1 : 50 (rabbit polyclonal)	MW	Overnight (about 15 h, 4°C)	Serotec
LMP-1	CS. 1–4	1 : 100 (mouse monoclonal)	—	Overnight (about 15 h, 4°C)	Dako
Egr-1	—	1 : 100 (rabbit polyclonal)	—	Nonovernight (1 h, RT)	Rockland

AID: activation-induced cytidine deaminase; LMP-1: latent membrane protein-1; Egr-1: early growth response-1; MW: microwave oven (for 1 min at high voltage and then for 10 min at low voltage); —: none; RT: room temperature.
